# Development of self-administered questionnaire on barriers, prescription practices, and guideline adherence of osteoporosis management among tertiary care clinicians: content validity and reliability analysis

**DOI:** 10.3389/fendo.2024.1393500

**Published:** 2024-09-06

**Authors:** Nur Khadijah Muhamad Jamil, Isa Naina Mohamed, Sabarul Afian Mokhtar, Juzaily Fekry Leong, Nur Azree Ferdaus Kamudin, Norliza Muhammad

**Affiliations:** ^1^ Department of Pharmacology, Faculty of Medicine, Universiti Kebangsaan Malaysia, Kuala Lumpur, Malaysia; ^2^ Department of Pharmacology, Faculty of Medicine, Universiti Sultan Zainal Abidin, Kuala Terengganu, Terengganu, Malaysia; ^3^ Department of Orthopedics, Faculty of Medicine, Universiti Kebangsaan Malaysia, Kuala Lumpur, Malaysia; ^4^ Department of Orthopedics, Faculty of Medicine, Universiti Sultan Zainal Abidin, Kuala Terengganu, Kuala Terengganu, Malaysia

**Keywords:** osteoporosis, barriers, practices, guideline, questionnaire, content validity, reliability

## Abstract

**Objective:**

This study outlined the development of the barriers, prescribing practices, and guideline adherence for osteoporosis management according to the Clinicians’ Osteoporosis Questionnaire (COQ) followed by an assessment of the content validity index and reliability test.

**Methods:**

The development of the COQ was performed in two stages. Stage I involved the development of the COQ, and stage II involved judgmental evidence and quantification of the questionnaire. Five panel experts related to the study area and five clinicians participated in the validity of the COQ assessment. Fifty clinicians took part in the reliability test evaluation by filling out the questionnaire twice at 2-week intervals. The content validity index (CVI) and content validity ratio (CVR) were analyzed using Microsoft Excel, while Cohen’s kappa statistic was used to determine the test–retest reliability using SPSS version 29.

**Results:**

Forty items and three domains, namely, barriers, prescribing practices, and guideline adherence for osteoporosis management, were identified in the COQ (version 4.0). The scale-level CVI (S-CVI/Ave) for every domain was above 0.9, which is considered acceptable. The CVRs for all the items were above 0.7, except for two items in the barrier domain and two items in the guideline adherence domain. Two items were revised to improve the clarity of the item, and other items were retained based on consensus among the expert panel. Between the test and retest, the reliability of individual items ranged from moderate to almost perfect for the barrier domain (k = 0.42–0.86), prescribing practice domain (k = 0.79–0.87), and guideline adherence domain (k = 0.46–1). None of the items had “fair” or “poor” agreement. Thus, the 40-item COQ (version 4.0) was finalized following the content and face validity analysis.

**Conclusions:**

Through an iterative process, the development and assessment of the COQ showed a high degree of content validity and reliability in measuring the barriers, prescribing practices, and guideline adherence among clinicians managing osteoporosis. Future studies should aim to further validate this instrument across different populations and settings, as well as explore methods to enhance its reliability and validity.

## Introduction

Osteoporosis is a chronic degenerative bone disease characterized by reduced bone density that increases susceptibility to fragility fractures, leading to substantial morbidity, mortality, and socioeconomic burden (1, 37, 38). The Asian Federation of Osteoporosis Societies has projected that there will be a 3.5-fold increase in osteoporotic hip fractures by 2050, from 6,000 to almost 21,000 fractures each year, costing over USD125 million (MYR540 million) annually in healthcare expenditure (2). Malaysia will have the highest expected rate of increase among countries in Southeast Asia due to the rapidly increasing aging population (2, 39). Thus, future fracture prevention strategies, which include optimizing osteoporosis management in clinical settings, are key to reducing this burden.

Osteoporosis is preventable and treatable, yet it remains underdiagnosed, undertreated, and undermanaged globally (3). The silent nature of this disease poses a significant challenge for clinicians to detect it early (4). Consequently, fragility fractures following mild falls or impacts are typically the first presenting symptom among patients with osteoporosis in tertiary care settings (4). Despite this, there is a paucity of studies focusing on the management of osteoporosis by tertiary clinicians. Most of the literature primarily focuses on primary care physicians’ role in managing osteoporosis (5–8). However, the unique challenges and opportunities in tertiary care settings warrant dedicated research attention, as tertiary care providers often serve as frontline clinicians for patients with fragility fractures. Moreover, compared to primary care settings, tertiary clinicians are well positioned to diagnose the underlying cause of fractures, as tertiary centers are usually well equipped with the necessary facilities, including the assessment of bone mineral density (BMD) using dual-energy X-ray absorptiometry (DXA), which is the gold standard for diagnosing osteoporosis (7). Following diagnosis, these patients also had greater opportunity for earlier initiation of anti-osteoporosis medication and provided interventions for fall prevention. The study by Boff et al. (9) highlighted that the most concerning gap in current practice among clinicians managing osteoporosis is that they tend to treat the fracture solely without further diagnosing the underlying cause of the fracture. As a result, they miss the opportunity to initiate antiresorptive drugs as early as possible. These drugs have been proven to be effective in increasing bone density and preventing further fractures (10). This gap in treatment is globally acknowledged as a crisis in osteoporosis patient care, which underscores the seriousness of this condition (9, 11, 12). While the occurrence of a previous fragility fracture almost doubles a patient’s future fracture risk if anti-osteoporosis medications are not started promptly, this condition will lead to a vicious cycle of recurrent fractures, often resulting in disability and premature death (13).

The effective management of osteoporosis among clinicians can be hindered by several factors. For example, barriers to osteoporosis care can significantly influence the efficacy of management strategies. These barriers may encompass a lack of awareness of current guidelines, variations in available pharmacotherapy, poor communication within the care team, and patient-related factors (3). Prescribing practices also play a pivotal role. The timely initiation of anti-osteoporotic medications, along with related vitamins and supplements, and the assessment of medication adherence could greatly enhance patient outcomes (10). In terms of guideline adherence, it is important to highlight measures for preventing osteoporosis and secondary fractures, appropriate diagnosis, and treatment follow-up to bridge the gap in osteoporosis management (8). However, it is noteworthy that previous studies have not documented in detail the validation and reliability of their questionnaires. Therefore, this study aimed to develop a valid and reliable self-administered questionnaire that can effectively capture the barriers, prescription practices, and adherence to guidelines among clinicians and will serve as a valuable tool for understanding and improving osteoporosis management in tertiary care settings, ultimately reducing the burden of this treatable condition. This study outlines the process involved in the development of the Clinicians’ Osteoporosis Questionnaire (COQ), as well as the methods employed to ascertain its content validity and test–retest reliability.

## Methods

### Study design

**Figure 1:**
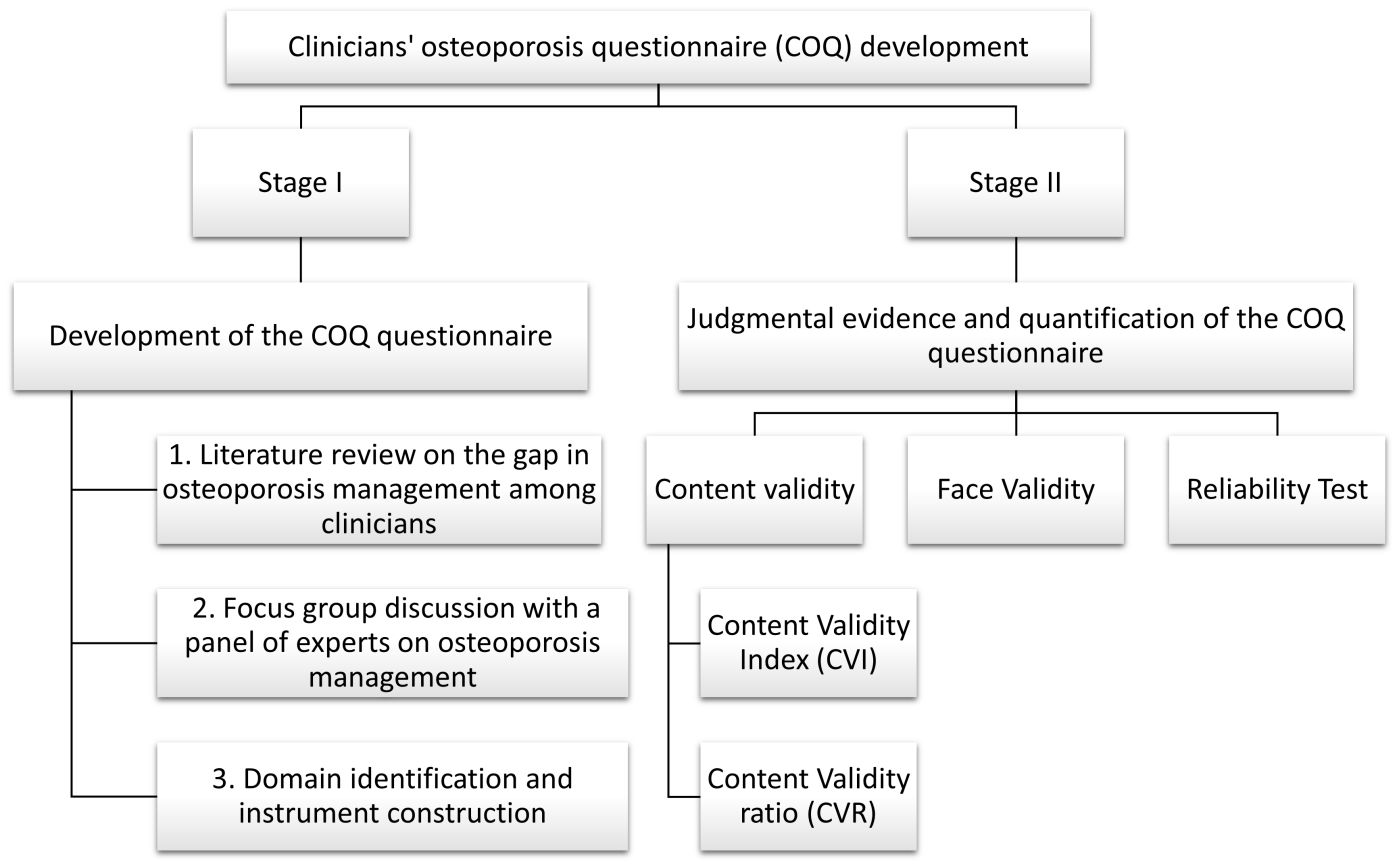
Summary of the development, validity, and reliability of the COQ. COQ, Clinicians’ Osteoporosis Questionnaire.

This cross-sectional study was conducted at the outpatient orthopedic department of tertiary healthcare institutions in Malaysia. The development, validation, and reliability assessment of the COQ were performed in a two-stage process, as outlined in **Figure 1**. Stage I involved instrument design and development, and Stage II involved obtaining judgmental evidence and quantifying the COQ, which included an analysis of content validity, face validity, and reliability. The panels of experts were invited to assess content validity through a judgmental sampling method based on their expertise, specialty, and credibility. For face validity, respondents were chosen using purposive sampling among orthopedic clinicians managing osteoporosis.

#### Stage I: development of the COQ

##### Item generation

The items for the COQ were generated through a thorough literature review that evaluated barriers, prescription practices, and guideline adherence to osteoporosis; the latest version of Malaysia’s Clinical Practice Guideline (CPG) of Osteoporosis 2022; and a focus group discussion conducted among osteoporosis subject matter experts. Given the limited research involving tertiary care clinicians, the items were derived from studies conducted on primary care physicians, supplemented with insights relevant to tertiary care settings, which include the following:

i. Literature review:

- Malaysian Primary Care Study (Tay et al., 2022): Identified knowledge, attitudes, practices, and barriers in osteoporosis management among primary care doctors.
- Singapore Primary Care Study (Derek et al., 2023): Evaluated osteoporosis guideline utilization and barriers to care among primary care physicians.
- Osteoporosis Medication Study (Yi et al., 2020): Assessed factors contributing to the initiation of osteoporosis medications post-hip fracture, as well as compliance and persistence with these medications.

ii. Clinical Practice Guidelines:

- The latest Malaysia CPG for Osteoporosis 2022 provided a framework for identifying essential practices and barriers relevant to osteoporosis management.

iii. Focus group discussion:

- Conducted with osteoporosis expert panel to identify additional barriers and practices specific to tertiary care settings.

Based on the results of the extensive literature review, the Osteoporosis CPG, and the discussion with experts, a conceptual framework was constructed, and a preliminary version of the COQ consisting of three domains (barriers, prescribing practices, and osteoporosis guideline adherence) was created.

#### Stage II: judgmental evidence and quantification of the COQ

This stage included three evaluations. The first was an assessment by expert panels, which focused on the content validity of each item in the COQ, specifically on relevance, clarity, and essentiality. Second, the face validity was evaluated via interviews and focus groups among orthopedic clinicians managing osteoporosis. Finally, the reliability of the questionnaire was evaluated among 50 orthopedic clinicians in a test–retest study.

##### Content validity

###### Expert panel

The number of experts recommended for content validation purposes can range from 2 to 20 individuals (14). To ensure that the consensus among experts represents their professional knowledge and comprehension of the subject matter rather than mere coincidence, it is recommended that a minimum of five individuals review the instruments. In this study, the content validation was determined by the following five members of the expert panels:

- one medical lecturer in bone and osteoporosis research,
- one medical lecturer in pharmacoepidemiology, and
- three orthopedic clinicians specializing in osteoporosis and fracture liaison services.

Experts were invited to participate in the content validity survey, which was sent via email. The email included a cover letter, the COQ, and an evaluation sheet asking for experts’ input on several aspects of each question in the tool, including 1) the importance of each question (relevance), 2) the clarity of each question (in terms of wording), 3) the necessity of each question (essentiality), and 4) recommendations for improvement of each question. A 4-point Likert scale was used for relevance, while a 3-point Likert scale was used for clarity and essentiality. The relevance scale includes 1 = not relevant, 2 = somewhat relevant, 3 = quite relevant, and 4 = very relevant, where the question is considered valid if the ratings are 2 and 3, while a rating of 1 is considered invalid. The clarity scale was 1= not clear, 2 = item needs some revision, and 3 = very clear. For the essentiality, it was 1 = not essential, 2 = useful but not essential, and 3 = essential.

###### Content validity evaluation

**Figure 2:**
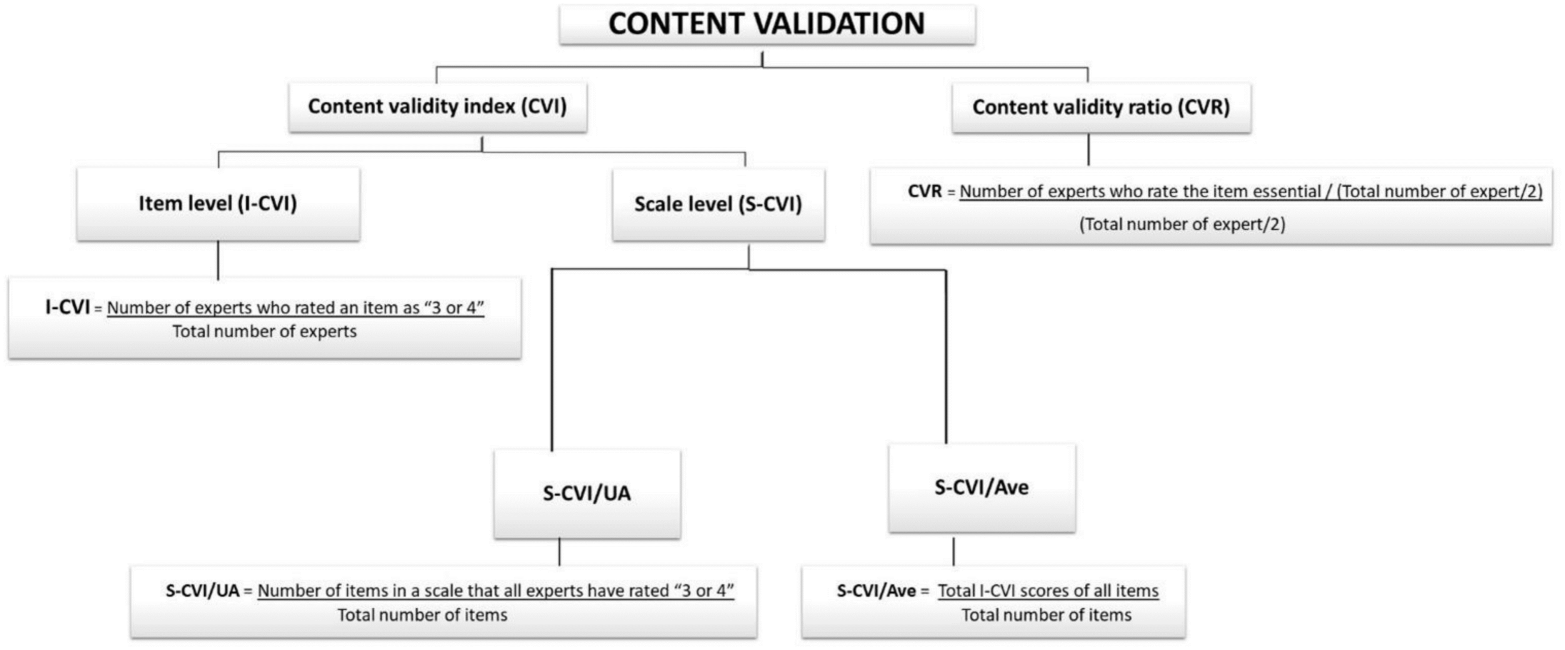
Summary of the quantitative analysis of content validity.

The COQ underwent thorough testing to confirm its validity. Validity refers to the degree to which an instrument accurately measures what it is supposed to measure (14). Consequently, several iterations were performed on the COQ to ensure that the survey was unambiguous and well defined and covered topics of importance to clinicians managing osteoporosis. In this study, content validity was quantitatively measured using the content validity index (CVI) for relevance and the content validity ratio (CVR) for the essentiality of each question. The calculation is summarized in **Figure 2**.

###### Content validity index

The CVI is a commonly used method for assessing content validity in newly developed instruments (15). It can be measured at both the item level (I-CVI) and the scale level (S-CVI). The 4-point Likert scale was used to rate each item (1 = not relevant, 2 = needs some revision, 3 = needs minor revision, and 4 = very relevant). The I-CVI is determined by dividing the number of experts who rated an item as 3 or 4 by the total number of experts (16). The I-CVI can range from 0 to 1. If the I-CVI is greater than 0.79, the item is considered relevant. If it falls between 0.70 and 0.79, the item requires revisions. An item with an I-CVI value below 0.70 was eliminated. When there are more than five experts, an I-CVI value of 0.78 is considered acceptable (16, 17).

The Scale-Content Validity Index (S-CVI) indicates the average score of the overall content validity scale. An S-CVI value of 0.8 or higher is generally considered acceptable (17). There are two methods for calculating the S-CVI:

- Universal Agreement (UA) among experts (S-CVI/UA) was calculated as the number of items on a scale that all experts rated as 3 or 4 (representing universal agreement) divided by the total number of items. The number of experts involved greatly affected the S-CVI/UA, in which the likelihood of obtaining a lower S-CVI is greater when there are more experts. This is because a UA score of 1 is assigned only when there is complete consensus among all experts on an item. If there was any disagreement, the UA score was set to 0.
- The average CVI (S-CVI/Ave) is the total I-CVI score of all the items divided by the total number of items (the average I-CVI score for all the items of all the experts).

###### Content validity ratio

The CVR is a measure of the essentiality of each item. The experts rated each item on a 3-point scale (1 = not essential, 2 = useful but not essential, and 3 = essential). Ratings of 1 and 2 signify “not essential” content, while ratings of 3 signify “essential” content (18). The CVR is calculated by subtracting half the total number of experts from the number of experts who rated an item as “essential” and then dividing this result by half the total number of experts (CVR = (ne(N/2)) (19, 20). The CVR ranges from −1 to 1, with a higher score indicating a higher level of agreement among panel members. A higher score signifies greater consensus among the expert panels regarding the necessity of the items. The minimum acceptable CVR for the six panels of experts involved was 0.99 (27). However, a CVR value of at least 0.78 is generally acceptable; if <0.78, the individual item should be revised or removed (28).

##### Face validity evaluation

Face validity can be explored both qualitatively (via a cognitive interview or a focus group discussion between researchers and a few respondents from the target population) and quantitatively (via feedback), which is measured on a Likert-scale survey. A cognitive interview is a method that helps individuals comprehend and respond to survey questions (21). It aims to gain insights into the thought process behind their responses and identifies potential issues. The question-and-answer model, which includes understanding, memory retrieval, decision-making, and response selection, predicts how individuals determine the level of detail needed for their answers. However, focus groups, which are informal discussions on a specific topic, were used in this study to gather participants’ understanding, opinions, and perspectives of the items in the questionnaire (22). In this study, face validity was qualitatively measured using a pilot group of five orthopedic medical officers who filled out the questionnaire and were encouraged to give comments, either written or oral, regarding the questionnaire. All the necessary information was noted.

##### Reliability test

The reliability of the questionnaire was evaluated in a test–retest study involving a sample of 50 orthopedic clinicians from tertiary care institutions in Malaysia. Participants were asked to complete the questionnaire on two separate occasions at 2-week intervals. The responses from the two administrations were compared, and Cohen’s kappa statistic was used to determine the test–retest reliability of each questionnaire item.

Cohen’s kappa statistic is a measure of the level of agreement between two raters who each classify items into mutually exclusive categories (23, 40). the study was extensively utilized in reliability analysis: It accounts for the possibility of agreement occurring by chance. There are two types of kappa—unweighted and weighted:

- Unweighted kappa: This type treats all disagreements equally. For example, if we have a scale from 1 to 5, a disagreement between 2 and 1 is treated the same as a disagreement between 5 and 1.
- Weighted kappa: This version of kappa is used for measuring agreement on ordered variables, where certain disagreements (e.g., lowest versus highest) can be weighted as more or less important (23). For example, on a scale from 1 to 5, a disagreement between 2 and 1 could be considered less serious than a disagreement between 5 and 1. In both cases, a higher kappa value indicates a higher level of agreement.

The strength of agreement was interpreted in line with the reference values provided by Landis and Koch (23). According to the scale, a Cohen’s k coefficient of less than 0.20 indicates poor agreement, 0.21–0.40 indicates fair agreement, 0.41–0.60 indicates moderate agreement, 0.61–0.80 indicates substantial agreement, and 0.81–1.00 represents almost perfect agreement. In this study, the unweighted k statistic was used to evaluate nominal variables, whereas the quadratic weighted k statistic was used to evaluate ordinal variables.

### Statistical analysis

For the content validity calculation, Microsoft Excel was used for data entry and tabulation of the CVI and CVR. The Cohen’s kappa test for the reliability of the questionnaire was conducted using IBM SPSS Statistics for Windows (version 29.0).

## Results

### Stage I: COQ revisions and development

From the COQ conceptual framework, three domains were identified: barriers, prescribing practices, and guideline adherence to osteoporosis management. The COQ underwent four rounds of revisions from the expert panels. **Table 1** summarizes the major amendments.

**Table 1:** Summary of major amendments of the COQ revision.

Version number	Reviewers	Total number of items in the beginning/total number of items in the end	Significant changes
Version 1	Five-member panel	60/26	-Changes of section 2 (Practice) to Prescribing Practice -Revised answer format from 3-point Likert scale to 4-point Likert scale for section 2: Practice
			-Removal of non-relevant CPG adherent items that focus on knowledge
Version 2	Five-member panel	26/38	-Addition of 12 items on CPG Adherent
Version 3	Five-member panel	38/40	-Analysis of pilot test done among orthopedic medical officer
			-Removal of 1 question in barrier section, removal of 2 questions from prescribing practice section, addition of 5 questions in CPG adherent section
Version 4	Five-member panel	40/40	-Analysis postrepilot test: -Rearrangement of questions according to domains in section 3, no other significant changes made

COQ, Clinicians’ Osteoporosis Questionnaire; CPG, Clinical Practice Guideline.

#### COQ revision results

In the first round, the expert panels evaluated the items for relevance and clarity. The second domain was initially regarded as “practice” but was changed to “prescribing practice” following discussions. The response format for prescribing practice was also changed from a 3-point scale to a 4-point Likert scale. The research team found that the items in the third domain (guideline adherence) were more suited to assessing knowledge rather than adherence to guidelines. As a result, all items in the third domain were eliminated, leaving out 26 of the initial 60 items (COQ version 1.0).

During the second round, 12 new items were developed from the latest version of Malaysia’s Osteoporosis CPG 2022. A dichotomous answer format (yes or no) was selected for the guideline adherence domain. Consequently, the total number of items increased to 38, up from the initial 26 in the second version (COQ version 2.0).

The third revision round took place after a pilot test of the 38-item questionnaire was conducted among 20 orthopedic medical officers involved in osteoporosis management at Hospital Canselor Tuanku Muhriz (HCTM), Cheras, Malaysia. The pilot test analysis led to the removal of one item from the barrier domain, two items from the prescribing practice domain, and the addition of five items to the guideline adherence domain, resulting in a total of 40 items in the questionnaire (COQ version 3.0).

For the fourth revision round, a repilot test was conducted with the 40-item questionnaire among 50 orthopedic clinicians managing osteoporosis. The analysis following the repilot test was satisfactory. A minor amendment was made where items were rearranged according to the subdomain in the guideline adherence domain. The final version of the questionnaire (COQ version 4.0), consisting of 40 items, was used in this study’s analysis. The summary of each domain, with a total of 40 items, is presented in **Table 2**.

**Table 2:** The domain, subdomain, and number of items of the COQ (version 4.0).

Domain	Subdomain	No. of item	Total items
Barriers	Knowledge	5	15
	Facilities	4	
	Patients	6	
Prescribing practice	Practice prescribing anti-osteoporosis medication	4	8
	Practice on follow-up the prescribed medication	4	
Guideline adherence	Preventive measures for osteoporosis	6	17
	Diagnosis	4	
	Treatment	7	
**Total no. of items**			**40**

COQ, Clinicians’ Osteoporosis Questionnaire.

#### COQ item development results

The domain concerning perceived barriers, which includes 15 items, was further categorized into three subdomains: barriers to knowledge of disease, facility-related barriers, and patient-related barriers. Out of 15 items, only six items were adopted from the study by Li et al. (7) and Choong et al. (8) (lack of doctor–patient time, inadequate knowledge, inaccessibility of pharmacotherapy at your practice, worry about side effects of the anti-osteoporotic medication, patients’ lower socioeconomic status, and patients’ refusal of screening). The remaining nine items were added to this study following the update from the latest version of the Osteoporosis CPG and the results from the expert recommendations of the subjects. This included the “silent” nature of the disease, which made it difficult to detect early, difficulty interpreting DXA scan results, inadequate knowledge of the different variations of calcium supplements and vitamin D, lack of choices of anti-osteoporotic medication at your hospital, lack of variation of calcium supplements and vitamin D at your hospital, difficulty integrating with another department (e.g., gynecology, geriatric, endocrinology, and primary care), inadequate staff to provide a better postfracture care program such as the Fracture Liaison Service (FLS), patients’ inadequate knowledge of osteoporosis, patient refusal to start anti-osteoporotic medication, and patient non-adherence to anti-osteoporotic medication. A 3-point Likert scale was used to measure the barriers to osteoporosis management in the barrier domain.

The prescribing practice comprised eight items with two subdomains, namely, practice on prescribing anti-osteoporosis medication (bone mineral consideration, history of fracture consideration, calcium, and vitamin D prescription) and practice on follow-up of the prescribed medication (compliance assessment, consideration to switching to other anti-osteoporosis medication if no improvement, scheduling DXA scans to monitor osteoporosis treatment, and recommendation of drug therapy when necessary). The study by Li et al. (7) served as a reference, but no items were directly adopted due to different contexts, as their study focused on primary care physicians. Consequently, the eight items in this domain were newly developed based on the most recent osteoporosis CPG, a literature review, and expert consensus. The response options employ an ordinal scale following the approach of Li et al. (7), with a 4-point Likert scale [never (0%), occasional (1%–50%), often (51%–99%), and always (100%)] to indicate the prescribing practice in osteoporosis management.

For the guideline adherence domain, 17 items were developed with three subdomains, including preventive measures for osteoporosis (eight items), diagnosis (four items), and treatment (seven items). The treatment subdomain differed from the prescribing practice domain, as the latter primarily emphasizes existing prescribing practices, which may or may not be included in the current guidelines. Conversely, the treatment subdomain focused mainly on treatment recommendations, as outlined in the latest Malaysia’s CPG, Osteoporosis, 2022 (24). This implies that while the prescribing practice domain reflects the prescribing practices that are currently being implemented (which could vary from the guidelines), the treatment subdomain strictly adheres to and focuses on the recommendations provided in the most recent guidelines. According to the study by Choong et al. (8), the research team found that the items regarding guideline utilization were too general, as there were only three questions: whether the participants read the latest guidelines, self-reported good knowledge, and self-reported good guideline utilization. Therefore, the research team decided to extract crucial practices from the latest osteoporosis CPG and finalized 17 items with a dichotomous answer format (yes or no) to measure the agreement with the statement.

### Stage II: diagnostic evidence and quantification of the COQ

#### Content validity analysis

Based on the expert panel’s judgment, all content validity (CVI and CVR) was calculated from the fourth version (three domains, 40 items) of the COQ and is shown in **Table 3** (barrier domain), **Table 4** (prescribing practice domain), and **Table 5** (guideline adherence domain).

**Table 3:** Summary items (I-CVI, CVR, and clarity) that were retained and revised for the barrier domain of the COQ (version 4.0).

Item		No. of the panel agree on the relevancy of the item	I-CVI	CVR	Clarity (mean)	Comments	Decision	New item after revision
B1	The “silent” nature of the disease made it difficult to detect it early	5	1	1	3		Retained	–
B2	Inadequate knowledge of current osteoporosis guidelines and medications	5	1	1	3		Retained	–
B3	Difficulty in interpreting DXA scan result	5	1	0.6	2.8	1/5 panel commented that this item is useful but not essential. Justification was made, as the DXA is often underutilized, and thus, interpreting DXA scans could pose significant challenges for clinicians. Therefore, this item is retained	Retained	–
B4	Inadequate knowledge of the different variations of calcium supplements and vitamin D	5	1	1	3		Retained	–
B5	Worry about the side effects of the anti-osteoporotic medication	5	1	1	3		Retained	–
B6	Lack of choices of anti-osteoporotic medication at your hospital	5	1	1	3		Retained	–
B7	Lack of variation of calcium supplements and vitamin D at your hospital	5	1	1	2.8		Retained	–
B8	Difficulty integrating with another department (e.g., gynecology, geriatric, endocrinology, and primary care)	5	1	0.6	2.6	1/5 panel commented that this item is useful but not essential. However, justification was made, as the multidisciplinary approach is crucially needed in managing osteoporosis, an aspect that is currently not well-established in practice. 3/5 of the panels suggest rephrasing the item. Thus, the item is revised and updated	Revised	Challenges in working together with other departments that are also involved in osteoporosis management
B9	Inadequate staff to provide a better postfracture care program such as Fracture Liaison Service (FLS)	5	1	1	3		Retained	–
B10	Lack of doctor–patient time	5	1	1	3		Retained	–
B11	Patients’ inadequate knowledge of osteoporosis	5	1	1	3		Retained	–
B12	Patient’s financial constraint due to socioeconomic status	5	1	1	3		Retained	–
B13	Patients’ refusal of screening	4	0.8	0.6	3	Only 1/5 panel commented that the item was somewhat relevant and not essential. CVR is not met. However, the consensus of the panels found it was one of the important barriers; thus, the item was retained	Retained	–
B14	Patient’s refusal to start anti-osteoporotic medication	5	1	1	2.8		Retained	–
B15	Patient’s non-adherence to anti-osteoporotic medication	5	1	1	3		Retained	–

I-CVI, item-level content validity index; CVR, content validity ratio; COQ, Clinicians’ Osteoporosis Questionnaire; DXA, dual-energy X-ray absorptiometry.

**Table 4:** The summary items (I-CVI, CVR, and clarity) that were retained and revised for the prescribing practice domain of the COQ (version 4.0).

Item		No. of the panel agree on the relevancy of the item	I-CVI	CVR	Clarity (mean)	Comments	Decision	New item after revision
P1	I consider bone mineral density when deciding to start anti-osteoporosis medication.	5	1	1	3	–	Retained	–
P2	I consider the history of fracture and clinical condition when deciding to start anti-osteoporosis medication.	5	1	1	3	–	Retained	–
P3	I prescribe calcium and vitamin D to all patients with fragility fractures.	5	1	1	3	–	Retained	–
P4	If both calcium supplements are available, I prefer to prescribe calcium carbonate over calcium lactate in non-End Stage Renal Failure (ESRF) patients.	5	1	1	3	–	Retained	–
P5	I assess osteoporosis medication compliance among patients during follow-up.	5	1	1	3	–	Retained	–
P6	I am considering changing to other osteoporosis medications if no bone mineral density improvement.	5	1	1	3	–	Retained	–
P7	I schedule the patient for the DXA scan accordingly to monitor the treatment given.	5	1	1	3	–	Retained	–
P8	If applicable, I recommend a drug holiday/stopping anti-osteoporotic therapy after a certain period.	5	1	1	3	-	Retained	-

I-CVI, item-level content validity index; CVR, content validity ratio; COQ, Clinicians’ Osteoporosis Questionnaire; DXA, dual-energy X-ray absorptiometry.

**Table 5:** The summary items (I-CVI, CVR, and clarity) that were retained and revised for the guideline adherence domain of the COQ (version 4.0).

Item		No. of the panel agree on the relevancy of the item	I-CVI	CVR	Clarity (mean)	Comments	Decision	New item after revision
G1	Do you identify risk factors for osteoporosis in advanced-age patients (>60 years old)?	5	1	1	3		Retained	–
G2	Do you emphasize exercise and physical therapy to prevent falls and injuries from falls?	5	1	1	3		Retained	–
G3	Do you advise patients on optimizing calcium and vitamin D intake?	5	1	1	3		Retained	–
G4	Do you perform multifactorial fall assessments and interventions in patients with risk of falls?	5	1	1	3		Retained	–
G5	Do you assess the risks of falls at least once a year in patients >65 years old?	5	1	1	2.8		Retained	–
G6	Do you provide interventions for fall prevention?	4	0.8	0.6	2.8	1/5 panel commented that the item is irrelevant and not essential. However, justification is made due to its importance in fracture prevention as highlighted in the latest osteoporosis CPG. Hence, the item is retained.	Retained	–
G7	For patients presenting with low trauma fracture, do you diagnose with osteoporosis despite the absence of BMD measurement?	5	1	1	3	2/5 of the panels recommend revising the item for improved readability. Therefore, the item is revised and retained.	Revised	Do you diagnose patients who present with low trauma fractures as having osteoporosis, even if there’s no BMD measurement available?
G8	Do you measure BMD in clinically diagnosed osteoporosis?	5	1	1	3		Retained	
G9	Do you recommend BMD measurement for all women above 65 and men above 70 yo?	5	1	1	3		Retained	–
G10	Do you perform blood tests or related investigations to exclude secondary causes of osteoporosis?	5	1	1	3		Retained	–
G11	Do you measure BMD to monitor treatment efficacy?	5	1	1	3		Retained	–
G12	Do you initiate anti-osteoporosis drugs without delay in patients with recent fractures (within the past 2 years)?	5	1	1	3		Retained	–
G13	Do you obtain renal function test before starting the bisphosphonate medications?	5	1	1	3		Retained	–
G14	Do you prescribe anti-resorptive (bisphosphonate or denosumab) to patients who are at high risk of osteoporosis-related fractures?	5	1	1	3		Retained	–
G15	Do you reassess the patient’s fracture risk after 3–5 years of bisphosphonate therapy?	5	1	1	3		Retained	–
G16	Do you consider anabolic agents (teriparatide or romosozumab) in patients with very high risk of fracture?	5	1	1	3		Retained	–
G17	Do you periodically monitor serum and urinary calcium in patients on activated vitamin D and calcium supplements?	5	1	1	3		Retained	–

I-CVI, item-level content validity index; CVR, content validity ratio; COQ, Clinicians’ Osteoporosis Questionnaire; CPG, Clinical Practice Guideline; BMD, bone mineral density.

The results showed that the I-CVI values, which indicate the relevance of individual items of all 40 items in the COQ (version 4.0), were beyond the acceptability cut-off point of 0.78. All items demonstrated an I-CVI value of 1, except for two items, one from the barrier domain (B13) and another from the guideline adherence domain (G6), both of which had an I-CVI of 0.8. In terms of the overall relevance of the questionnaire, represented by the S-CVI values, the results also exceeded the cut-off point of 0.8, with the S-CVI/UA (overall) being 0.95 and the S-CVI/Ave (overall) being 0.99. The S-CVI/UA and S-CVI/Ave per domain were also calculated and showed acceptable values (>0.8).

The CVR calculations indicated that four items (B3, B8, B13, and G6) out of the 40 items in the COQ did not meet the acceptable value of 0.99. This value was set based on the evaluations of a panel consisting of five experts as suggested by Lawshe et al. Each of these items had a CVR value of 0.6. While non-essential items can potentially be removed, in this instance, they were retained based on the recommendations and consensus of the expert panels. A detailed summary of the items across all domains is presented in **Table 6**.

**Table 6:** The summary of the items for all domains in COQ (4.0) following the content validity analysis.

Domains	No. of items	No. of items revised	No. of items deleted	No. of items retained
Barriers	15	1	–	15
Prescribing practice	8	–	–	8
Guideline adherence	17	1	–	17
**Total**	**40**	**2**	**-**	**40**

The bold values in the last row represent the total number of items after revisions, deletions, and retention across all domains in the COQ version 4.0.

The clarity of the fourth version of the COQ was evaluated by five raters using a 3-point Likert scale, where 1 signifies “not clear”, 2 represents “somewhat clear”, and 3 denotes “very clear”. The mean clarity scores for individual items were calculated, and the results varied between 2.60 and 3.00. A total of 34 items, accounting for 85% of the items, were classified as “very clear”, with an average score of 3.00. Five items received an average clarity score of 2.80, while one scored 2.60. The overall clarity mean score for the fourth version of the COQ was determined to be 2.97.

#### Face validity analysis

The five-member pilot group qualitatively confirmed the face validity of the COQ. All participants in the group unanimously agreed on the relevance, essentiality, and clarity of the items included in the questionnaire, which they found to be straightforward and easy to comprehend.

#### Reliability test analysis

##### Test–retest reliability

Of the 60 initial respondents, 83% (50) of the clinicians successfully completed the questionnaire on two separate occasions at 2-week intervals. Most of the respondents were male doctors (70%, 35), and the mean age of the respondents was 33.76 (4.35) years. Most clinicians who participated in the survey were of Malay ethnicity, represented by 80% (40). The detailed characteristics of the clinicians who participated in the survey are presented in **Table 7**.

**Table 7:** Characteristics of the study participants.

Characteristics	(%)	(n)	Mean (SD)
Age			
20–30	20	10	33.76 (4.35)
31–40	70	35	
41–50	10	5	
Gender			
Male	70	35	
Female	30	15	
Ethnicity			
Malay	80	40	
Chinese	10	5	
Indian	10	5	
No of years working as doctor			
1–10	68	34	8.76 (4.35)
11–20	28	14	
21–30	4	2	
No. of years working in the orthopedic department			
1–10	86	43	6.18 (4.38)
11–20	12	6	
21–30	2	1	
Estimated number of osteoporosis patients seen in a month			
<10	34	17	
11–50	62	31	
>50	4	2	

The reliability of the three domains of the questionnaire was assessed, and the results are presented in **Tables 8**–**10**. The overall kappa agreement ranged from 0.42 to 1, with no items having kappa values less than 0.40, indicating no “fair” or “poor” agreement in the barrier domain; the kappa agreement values ranged from 0.42 to 0.86. Seven items demonstrated substantial agreement, five items showed moderate agreement, and three items exhibited almost perfect agreement. The item pertaining to the limited choices of anti-osteoporotic medication at the hospital had the highest agreement (k = 0.86). In contrast, the item regarding patients’ inadequate knowledge of osteoporosis had the lowest agreement (k = 0.42). The percentage of agreement for this domain ranges from 70.0% to 94.0%.

**Table 8:** Cohen’s kappa (k) coefficient for the barrier domain.

Item no.	Detail item	Percentage agreement (%)	Kappa coefficient (weighted)	Interpretation
B1	The “silent” nature of the disease made it difficult to detect it early	94	0.615	Substantial agreement
B2	Inadequate knowledge of current osteoporosis guidelines and medications	70	0.431	Moderate agreement
B3	Difficulty in interpreting DXA scan result	84	0.726	Substantial agreement
B4	Inadequate knowledge of the different variations of calcium supplements and vitamin D	84	0.836	Almost perfect agreement
B5	Worry about the side effects of the anti-osteoporotic medication	86	0.754	Substantial agreement
B6	Lack of choices of anti-osteoporotic medication at your hospital	86	0.861	Almost perfect agreement
B7	Lack of variation of calcium supplements and vitamin D at your hospital	86	0.822	Almost perfect agreement
B8	Difficulty integrating with another department (e.g., gynecology, geriatric, endocrinology, and primary care)	80	0.675	Substantial agreement
B9	Inadequate staff to provide a better postfracture care program such as Fracture Liaison Service (FLS)	76	0.548	Moderate agreement
B10	Lack of doctor–patient time	82	0.656	Substantial agreement
B11	Patients’ inadequate knowledge of osteoporosis	80	0.416	Moderate agreement
B12	Patient’s financial constraint due to socioeconomic status	74	0.44	Moderate agreement
B13	Patients’ refusal of screening	78	0.741	Substantial agreement
B14	Patient’s refusal to start anti-osteoporotic medication	82	0.763	Substantial agreement
B15	Patient’s non-adherence to anti-osteoporotic medication	78	0.603	Moderate agreement

DXA, dual-energy X-ray absorptiometry.

**Table 9:** Cohen’s kappa (k) coefficient for the prescribing practice domain.

Item no.	Detail item	Percentage agreement (%)	Kappa coefficient (weighted)	Interpretation
P1	I consider bone mineral density when deciding to start anti-osteoporosis medication.	84	0.813	Almost perfect agreement
P2	I consider the history of fracture and clinical condition when deciding to start anti-osteoporosis medication.	86	0.804	Substantial agreement
P3	I prescribe calcium and vitamin D to all patients with fragility fractures.	82	0.787	Substantial agreement
P4	If both calcium supplements are available, I prefer to prescribe calcium carbonate over calcium lactate in non-ESRF patients.	86	0.825	Almost perfect agreement
P5	I assess osteoporosis medication compliance among patients during follow-up.	88	0.872	Almost perfect agreement
P6	I am considering changing to other osteoporosis medications if no bone mineral density improvement.	84	0.803	Substantial agreement
P7	I schedule the patient for the DXA scan accordingly to monitor the treatment given.	90	0.841	Almost perfect agreement
P8	If applicable, I recommend a drug holiday/stopping anti-osteoporotic therapy after a certain period.	82	0.827	Almost perfect agreement

DXA, dual-energy X-ray absorptiometry.

**Table 10:** Cohen’s kappa (k) coefficient for the guideline adherence domain.

Item no.	Detail item	Percentage agreement (%)	Kappa coefficient (weighted)	Interpretation
Item 1	Do you identify risk factors for osteoporosis in advanced-age patients (>60 years old)?	92	0.459	Moderate agreement
Item 2	Do you emphasize exercise and physical therapy to prevent falls and injuries from falls?	96	0.728	Substantial agreement
Item 3	Do you advise patients on optimizing calcium and vitamin D intake?	100	1	Almost perfect agreement
Item 4	Do you perform multifactorial fall assessments and interventions in patients with risk of falls?	82	0.629	Substantial agreement
Item 5	Do you assess the risks of falls at least once a year in patients >65 years old?	86	0.719	Substantial agreement
Item 6	Do you provide interventions for fall prevention?	88	0.757	Substantial agreement
Item 7	For patients presenting with low trauma fracture, do you diagnose with osteoporosis despite the absence of BMD measurement?	92	0.769	Substantial agreement
Item 8	Do you measure BMD in clinically diagnosed osteoporosis?	86	0.645	Substantial agreement
Item 9*	Do you recommend BMD measurement for all women above 65 and men above 70 yo?	89	0.724	Substantial agreement
Item 10	Do you perform blood tests or related investigations to exclude secondary causes of osteoporosis?	84	0.663	Substantial agreement
Item 11	Do you measure BMD to monitor treatment efficacy?	94	0.788	Substantial agreement
Item 12	Do you initiate anti-osteoporosis drugs without delay in patients with recent fractures (within the past 2 years)?	92	0.793	Substantial agreement
Item 13	Do you obtain renal function test before starting the bisphosphonate medications?	92	0.816	Almost perfect agreement
Item 14	Do you prescribe anti-resorptive (bisphosphonate or denosumab) to patients who are at high risk of osteoporosis-related fractures?	94	0.805	Substantial agreement
Item 15	Do you reassess the patient’s fracture risk after 3–5 years of bisphosphonate therapy?	96	0.811	Almost perfect agreement
Item 16	Do you consider anabolic agents (teriparatide or romosozumab) in patients with very high risk of fracture?	86	0.72	Substantial agreement
Item 17	Do you periodically monitor serum and urinary calcium in patients on activated vitamin D and calcium supplements?	88	0.755	Substantial agreement

BMD, bone mineral density.

In the prescribing practice domain, the kappa agreement values ranged from 0.79 to 0.87. Most items (5) displayed almost perfect agreement, and three items showed substantial agreement. The item related to assessing patient compliance with osteoporosis medication during follow-up had the highest agreement (k = 0.86), while the item about prescribing calcium and vitamin D to all patients with fragility fractures had the lowest agreement (k = 0.79). The percentage of agreement for this domain varied from 82.0% to 90.0%.

In the guideline adherence domain, the kappa agreement values ranged from 0.46 to 1. Twelve items demonstrated substantial agreement, three items showed almost perfect agreement, and one item exhibited moderate agreement. The item about advising patients on optimizing calcium and vitamin D intake had the highest agreement (k = 0.86), while the item concerning identifying risk factors for osteoporosis in advanced-aged patients (>60 years old) had the lowest agreement (k = 0.79). The percentage of agreement for this domain ranged from 82.0% to 100.0%.

## Discussions

This study aimed to assess the content validation and reliability of a newly developed questionnaire on barriers, prescribing practices, and guideline adherence among clinicians managing osteoporosis (COQ). An approach suggested by Khosla and Shane (11) that has been widely used mainly in medical and nursing research was employed in this study. Content validity is an important quality indicator of an instrument’s validity. The researchers developed the COQ containing a total of 60 items in four domains at the first stage and ultimately retained 40 items for postface validity and pilot test analysis in the COQ version 4.0.

In accordance with the guidelines proposed by Khosla and Shane (11), which had been extensively utilized in this study area, a panel of five experts was selectively assembled to oversee the questionnaire development process and make necessary adjustments to the instrument. The panel comprised experts with a robust background in bone research, pharmacoepidemiology, and osteoporosis management. A medical lecturer with extensive experience in bone research was included, providing valuable insights into the development and refinement of the questionnaire. Additionally, three orthopedic surgeons were recruited, two of whom specialized in postosteoporotic fracture management and Fracture Liaison Service implementation. Their expertise allowed for the identification of gaps in current practice, providing a unique perspective that would otherwise be unattainable. In addition, an expert in clinical pharmacoepidemiology participated, providing guidance on question selection and formulation, ensuring item relevance and accuracy, and assisting in choosing appropriate answer options. This strategic recruitment not only strengthens our research methodology but also enriches the validity and reliability of our findings while shedding light on areas for improvement in current practice. While the panel’s expertise significantly contributes to the development and refinement of the instrument, it is crucial to note the potential bias due to the subjective nature of the panel expert’s feedback (15). This subjectivity could introduce a degree of bias into the process, which may influence the interpretation and revision of items in the instrument. To mitigate this risk, achieving consensus among panel experts is of paramount importance because we can harness their collective wisdom while minimizing the impact of individual biases, thereby ensuring the overall validity and reliability of the instrument (25).

### Discussion on CVI

In this study, the CVI was utilized as a key tool for refining the COQ (version 4.0). This approach is consistent with methodologies used in other studies. The CVI, supplemented by feedback and comments from the expert panel, served as a critical judgment tool to enhance the content validity indices. Both the item-level content validity index (I-CVI) and the scale-level content validity index (S-CVI) were considered in this process. The S-CVI provides an average score of the overall content validity scale, offering a comprehensive view of the instrument’s validity (14, 18). An I-CVI of 0.78 or higher is considered excellent. The I-CVIs of all the items in the COQ ranged from 0.80 to 1.00, with only two items having an I-CVI of 0.8, indicating high content validity. There were differing opinions among the panel regarding two items (both with I-CVI: 0.8): “Patient’s refusal of screening” in the barrier domain and “Do you provide interventions for fall prevention?” from the guideline adherence domain. These differences could be attributed to the diverse backgrounds and experiences of the experts (26). For instance, an expert with a clinical background may view these items as crucial elements in osteoporosis management, as they witnessed the situations in their clinical practice, while a non-clinical expert may not have had the same experiences, hence considering the items irrelevant. The minimum acceptable S-CVI ranges from 0.80 to 0.90 (17). In this study, two values were calculated: the Universal Agreement (S-CVI/UA = 0.95) and the average (S-CVI/Ave = 0.99). Both scores indicated excellent overall content validity of the COQ. This suggests that the individual items developed were important and relevant for measuring barriers, prescribing practices, and guideline adherence among clinicians managing osteoporosis.

The CVR was used to assess the quality of the items in the COQ. A total of 36 items achieved an excellent CVR value of 1, indicating that most of the raters considered these items to be essential (14). This resulted in an overall content validity score of CVR = 0.96. However, four items received a CVR of 0.6, suggesting that they were not considered essential by some of the panels. These items included three from the barrier domain [“Difficulty in interpreting DXA scan results”, “Difficulty integrating with another department (e.g., gynecology, geriatric, endocrinology, and primary care)”, and “Patients’ refusal of screening”] and one from the guideline adherence domain (“Do you provide interventions for fall prevention?”).

The differing opinions among the panel regarding these four items could be attributed to the interpretation of available evidence, as some experts may interpret the data to suggest that these items are significant barriers or crucial for guideline adherence in osteoporosis management, while others may not see a strong enough link. These differing perspectives underscore the complexity of developing a questionnaire and highlight the importance of achieving consensus among panel experts (27, 28).

The clarity of the COQ appears to be straightforward and comprehensible, as evidenced by 85% of the items being rated as “very clear”. This clarity is crucial because it allows respondents to answer the questionnaire with ease, which in turn ensures the reliability and validity of their responses (29). The qualitative validation of the face validity of the COQ in the pilot group further supported this conclusion. The unanimous agreement among the participants on the relevance, essentiality, and clarity of the items in the questionnaire indicates that the instrument is well designed. However, it is important to note that while these initial results are promising, they are not definitive. As a useful initial step, face validity is a subjective measure and does not guarantee overall validity because it is based on apparent relevance, not on a rigorous statistical examination of the measurement properties of the scale (30). Therefore, further validation studies using more robust statistical methods are necessary to confirm these initial findings and to assess other forms of validity (e.g., construct validity and criterion validity) and reliability (31, 32). The COQ is expected to offer valuable insights and reliable sources for clinicians managing osteoporosis in tertiary care settings that could lead to more effective management strategies and prevention of fractures, which in turn improve patient outcomes.

#### Reliability testing

In this study, the reliability of the questionnaire was assessed using test–retest reliability analysis. For the Likert scale items in the barriers and prescribing practice domains, a quadratic weighted kappa was employed. The quadratic weighted kappa is an extension of Cohen’s kappa that considers the severity of disagreements, making it particularly useful for ordinal scales where disagreements between categories that are farther apart are more serious than disagreements between adjacent categories (33). For the guideline adherence domain, Cohen’s kappa coefficient was used instead of the intraclass correlation coefficient (ICC) due to the dichotomous nature of the questionnaire responses (32). Cohen’s kappa statistic is a type of reliability coefficient that measures the degree of agreement between two different evaluations in questionnaires with dichotomous variables (34). The results showed that the kappa values were almost perfect for the prescribing practice domain (0.82), while the barriers and guideline adherence domains showed substantial agreement (0.66 and 0.74, respectively). The overall average kappa coefficient of the questionnaire was 0.73, indicating substantial agreement.

The substantial agreement in the barrier domain suggests that clinicians are facing similar challenges in their practice. The lack of choices of anti-osteoporotic medication at hospitals, for instance, could be a systemic issue that needs to be addressed at a policy level. This shared recognition of barriers could serve as a call for healthcare administrators and policymakers to improve the availability of medication options in tertiary care settings (8). In the prescribing practice domain, most items displayed almost perfect agreement and a high level of consistency among clinicians in their views on prescribing practices. This could be interpreted as a collective agreement on best practices for prescribing medication for osteoporosis, reflecting a standardized approach to treatment. This uniformity in prescribing practices is beneficial because it ensures that patients receive consistent care regardless of their healthcare provider (9). The substantial agreement in the guideline adherence domain reflects a common consensus among clinicians about the importance of adhering to guidelines in osteoporosis management. The high level of agreement in advising patients on optimizing calcium and vitamin D intake highlights the universal acknowledgment of this aspect in managing osteoporosis (35). This shared understanding underscores the importance of guideline adherence in ensuring effective and standardized care for osteoporosis patients. The absence of any “fair” or “poor” agreement across all domains further highlights the reliability of the questionnaire, with most items demonstrating substantial to almost perfect agreement. However, it is worth noting that even though the kappa values were generally high, there were variations within each domain. This suggests that while the questionnaire is generally reliable, there may be specific items that could benefit from further refinement to improve their clarity and ensure that they are accurately capturing the constructs they are intended to measure. In addition, direct comparison of these results with those of prior studies was difficult due to differences in the context of the questions and type of response alternatives used in the questionnaire. Moreover, the repeatability of most questionnaires used in previous studies related to clinicians managing osteoporosis has not been well statistically documented.

#### Application of COQ in clinical settings

The COQ involves identifying barriers, prescribing practices, and guideline adherence among clinicians managing osteoporosis. By systematically assessing these domains, applying the COQ in clinical settings can reveal critical gaps and challenges faced by healthcare providers, enabling the development of targeted interventions. For instance, identifying barriers such as inadequate knowledge of osteoporosis guidelines and medications, difficulty in interpreting DXA scan results, and patient-related factors like non-adherence to medication can help design educational programs tailored to clinicians’ needs. Similarly, understanding prescribing practices and adherence to guidelines can highlight areas where clinicians deviate from recommended protocols, facilitating the identification of gaps in practice and addressing areas needing improvement.

The legitimacy of the COQ as an effective tool in osteoporosis management is underscored by our recent study comparing barriers and guideline adherence between standard tertiary care and FLS-accredited hospitals (36). The COQ successfully highlighted important gaps and significant differences in guideline adherence between these settings. FLS settings were more likely to initiate timely treatment, monitor patients effectively using BMD assessments, and consider anabolic agents for high-risk patients. These insights demonstrate that the COQ is effective not only in identifying critical areas needing improvement but also in driving the development of interventions that enhance osteoporosis management practices and promote evidence-based policies.

Moreover, the COQ serves as a self-audit tool for clinicians. By simply reading and answering the straightforward and informative questions in the COQ, clinicians can self-assess how well they are practicing and adhering to osteoporosis guidelines. This immediate feedback can help clinicians identify areas for improvement in their clinical practice, making the COQ a practical and beneficial tool for continuous self-improvement and adherence to best practices.

As the COQ targets clinicians in tertiary centers, who are at the frontline of managing fragility fracture patients, it specifically addresses a prioritized group. This approach may improve discharge care plans for osteoporotic fracture patients as tertiary clinicians become more aware of and adhere to guidelines for secondary fracture prevention. These comprehensive care plans can then be continued in primary care settings, enhancing continuity of care. The integration with primary care also helps reduce the burden caused by the lack of time between doctors and patients in tertiary hospitals.

#### Targeted interventions and policy changes

Based on COQ findings, educational programs can be designed to address knowledge gaps among clinicians. For example, workshops or online courses can be developed to improve understanding of current osteoporosis guidelines and effective use of DXA scans.

If the COQ identifies systemic issues such as limited medication options or insufficient time for patient interactions, healthcare institutions can strategically revise policies to elevate bone health as a national priority, addressing gaps not currently listed in the National Health Agenda 2016–2025 (36). Such policy revisions could lead to allocating additional resources, ensuring a broader availability of osteoporosis medications, and granting clinicians more time for comprehensive patient consultations. Additionally, this approach could promote the wider adoption of FLS in hospitals, further enhancing the standard of care for osteoporosis management.

Recognizing barriers to patient adherence can lead to developing comprehensive adherence programs, including patient education, regular follow-ups, and the application of reminder systems to enhance medication compliance.

#### Impact on patient outcomes, clinician education, and healthcare resource allocation

Implementing the COQ in clinical settings has the potential to improve patient outcomes significantly. By ensuring that clinicians are well-informed about the latest guidelines and have access to necessary resources, patients are more likely to be diagnosed and receive timely and appropriate treatment and fall prevention measures, reducing the risk of fractures and other complications associated with osteoporosis. Clinician education is another critical area where the COQ can substantially impact. Continuous professional development based on COQ findings ensures clinicians stay updated with the latest advancements and best practices in osteoporosis management, ultimately leading to higher quality of care. Furthermore, the COQ serves as critical evidence-based support for healthcare resource allocation. By identifying resource-lacking areas, the COQ enables healthcare institutions to allocate funds more efficiently, ensuring that critical needs are met and resources are used effectively to improve overall patient care.

#### Integration with clinical workflows and electronic health records

Integrating the COQ with clinical workflows and electronic health record (EHR) systems makes it easier to use in real-world settings. Electronic integration allows for smooth data collection, analysis, and reporting, helping clinicians incorporate COQ assessments into their routine practice.

For example, the COQ can be added to EHRs for regular assessments, with automated prompts and reminders to ensure consistent use. The following outlines how the COQ can be effectively embedded into EHR systems: in the barrier domain, questions such as “Inadequate knowledge of current osteoporosis guidelines and medications” can be embedded into EHRs. When a clinician fills out a patient’s EHR, they could be prompted to answer questions about their knowledge and comfort level with current guidelines. If a clinician indicates a lack of knowledge, the system can automatically provide links to educational resources or suggest participation in upcoming workshops. In the prescribing practices domain, questions such as “I assess osteoporosis medication compliance among patients during follow-up” can be embedded into EHRs to prompt clinicians during patient visits. This can ensure that medication adherence is regularly checked and recorded in the patient’s health records. The EHR can also generate alerts if a patient is due for a follow-up DXA scan or if there are inconsistencies in medication adherence, allowing the clinician to address these issues promptly. In the guideline adherence domain, questions like “Do you measure BMD in clinically diagnosed osteoporosis?” can be integrated into EHRs to ensure clinicians adhere to guidelines. The EHR can include checklists that clinicians must complete during patient consultations, ensuring all necessary diagnostic and treatment steps are followed according to the latest guidelines. This can standardize care and ensure no critical steps are overlooked.

Additionally, EHR integration can enable real-time data analysis, providing immediate feedback to clinicians and administrators​. This can help identify trends and patterns, allowing for quick adjustments to management strategies and policies to improve patient outcomes​.

In summary, the COQ provides a strong framework for improving osteoporosis management in clinical settings. Its use can lead to targeted interventions, better clinician education, efficient healthcare resource allocation, and easy integration with clinical workflows and EHR systems, ultimately improving patient care and outcomes. Additionally, as a self-audit tool, it helps clinicians continually evaluate and improve their adherence to osteoporosis management guidelines. By targeting clinicians in tertiary centers, the COQ enhances secondary fragility fracture prevention in primary care settings and reduces time constraints in tertiary hospitals.

### Strengths and limitations

To the best of our knowledge, this is the first study on the development of a questionnaire that is specifically designed for clinicians managing osteoporosis in a tertiary care setting, as well as an in-depth discussion of the content validity and reliability of the questionnaire. However, this study has several limitations. While this study focused on content validity and test–retest reliability, other forms of validity (such as construct or criterion validity) were not assessed. These additional measures could provide a more comprehensive understanding of the questionnaire’s validity. The feedback from the pilot group, although valuable, represents a small sample size. To ensure broader applicability of the questionnaire, it would be beneficial to expand the evaluation to include a larger and more diverse group of participants. This would provide additional insights and help ensure that the questionnaire is generalizable across different contexts. Future research should aim to address these limitations by assessing other forms of validity, using larger and more diverse samples, and refining the questionnaire design. Additionally, factors such as potential biases in responses, cultural and geographical variations in osteoporosis management practices, and external validation of the questionnaire could also be considered to further enhance the robustness and applicability of the newly developed questionnaire, thereby contributing to better management of osteoporosis in various clinical settings.

## Conclusion

This study provides valuable insights into the content validity and reliability of the COQ and demonstrates its effectiveness in assessing barriers, prescribing practices, and guideline adherence among clinicians in tertiary care settings. Future research should aim to further validate this tool across diverse populations and settings and explore ways to enhance its reliability and validity. The COQ is a promising tool for practical application at various levels, potentially enhancing osteoporotic patient care and outcomes.

## Data Availability

The original contributions presented in the study are included in the article/supplementary material. Further inquiries can be directed to the corresponding author.
